# Social risk to infant: The role of kin for maternal visual monitoring in Tibetan macaques

**DOI:** 10.1002/ece3.11626

**Published:** 2024-06-25

**Authors:** Shen‐Qi Liu, Hong‐Wei Tian, Sen Ren, Wen‐Kai Sun, Peng‐Lai Fan, Dong‐Po Xia, Bing‐Hua Sun, Jin‐Hua Li, Xi Wang

**Affiliations:** ^1^ School of Resources and Environmental Engineering Anhui University Hefei China; ^2^ International Collaborative Research Center for Huangshan Biodiversity and Tibetan Macaque Behavioral Ecology Hefei China; ^3^ School of Life Sciences Anhui University Hefei China; ^4^ Key Laboratory of Ecology of Rare and Endangered Species and Environmental Protection (Guangxi Normal University) Ministry of Education Guilin China; ^5^ Guangxi Key Laboratory of Rare and Endangered Animal Ecology, College of Life Sciences Guangxi Normal University Guilin China; ^6^ School of Life Sciences Hefei Normal University Hefei China

**Keywords:** kin, maternal visual monitoring, social risk, Tibetan macaque

## Abstract

Maternal monitoring of conspecifics is a crucial anti‐predator strategy that also protects infants against risks within the social group. This study examines how maternal characteristics, infant characteristics, mother–infant distance, and the social environment affect maternal monitoring behaviors in free‐ranging Tibetan macaques (*Macaca thibetana*). We observed 12 females with infants and analyzed their visual monitoring patterns. Our findings indicate that maternal rank significantly influences the time allocated to maternal visual monitoring, higher‐ranking mothers spending less time than lower‐ranking mothers. Maternal experience also played a role in monitoring strategies. Differences in monitoring strategies were observed based on maternal experience: first‐time mothers (primiparity) engaged in longer but less frequent monitoring sessions compared to experienced mothers (multiparity). The time and frequency of maternal monitoring decreased as infants aged, and mothers with male infants showed higher levels of monitoring than those with female infants. The distance between mother and infant also affected visual monitoring behavior, with mothers increasing their monitoring levels when infants were nearby (1–5 m), rather than within reach (0–1 m) or beyond nearby (>5 m). Additionally, the presence of kin and non‐kin influenced monitoring: as the number of nearby kin increased, monitoring levels decreased, while the presence of more non‐kin males led to an increase in monitoring time, and higher‐ranking non‐kin neighbors increased the frequency of monitoring. These results suggest that Tibetan macaque mothers can adapt their visual monitoring to the social risks faced by their infants, adjusting their strategies to their status and the needs of their offspring.

## Graphical abstract

In this study, we investigated how the effects of maternal characteristics, infant characteristics, mother–infant distance, and social environment on maternal monitoring behaviors in a group of free‐ranging Tibetan macaques. Our results indicate that Tibetan macaque mothers possess the ability to adapt their level of visual monitoring in response to the social risks faced by their infants, and can adjust their monitoring strategies to their own and infant conditions.

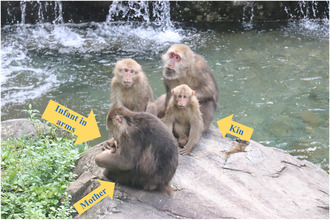

## 1 INTRODUCTION

Animals can acquire diverse ecological and social information (Kutsukake, 2007) and detect and avoid potential threats to themselves and their offspring (Kutsukake, 2006; Treves et al., 2003; Vine, 1971) by monitoring their surroundings. While individual visual monitoring has been investigated as an anti‐predator strategy (Pulliam, 1973; Elgar, 1989; Lima & Dill, 1990; Caro, 2005) revealing a negative relationship between group size and individual vigilance (Clutton‐Brock et al., 1999; Lima, 1995), recent studies have increasingly emphasized the importance of monitoring conspecifics—known as social vigilance (Kutsukake, 2007). In many highly social primate species, individual vigilance was positively correlated with group size (e.g., mustached tamarins, *Saguinus mystax*: Stojan‐Dolar & Heymann, 2010; ursine colobus monkeys, *Colobus vellerosus*: Teichroeb & Sicotte, 2012).

Monitoring conspecifics often relates to risks posed by different individuals (Treves, 2000). The degree of threat from conspecifics potentially varies when interacting with different group members, influencing vigilance levels based on relationship quality (Busia et al., 2019; Kutsukake, 2006). Individuals are expected to increase their vigilance when nearby conspecifics pose a higher threat (Gaynor & Cords, 2012). If there is a higher proportion of kin or allies among neighbors, however, they can rely on the vigilance of potential companions to reduce social risk (Iki & Kutsukake, 2021). Some studies found that individuals decrease their level of vigilance when kin are present nearby or when the majority of neighbors are kin (blue monkeys, *Cercopithecus mitis*: Gaynor & Cords, 2012; Japanese macaques, *Macaca fuscata*: Iki & Kutsukake, 2021). Relatives exhibit lower levels of aggression and are more inclined to form alliances against other aggressors, potentially leading to a reduction in stress levels and a decrease in vigilance (Cords, 2002; Silk et al., 2003; Watts, 1998).

Infanticide is an extreme manifestation of sexual conflict and has been documented in numerous primate species (Struhsaker & Leland, 1987). Sexual selection theory suggests that males would benefit from infanticide (van Schaik & Kappeler, 1997). However, infanticide is costly for females, and as a consequence, females have a wide variety of strategies (Lukas & Huchard, 2014), including territorial defense (Wolff & Peterson, 1998), heightened vigilance (Breedveld et al., 2019), and aggressive responses toward intruders (Elwood et al., 1990). In species without reported infanticide, mothers still exhibit protective strategies, such as Bornean orangutans (*Pongo pygmaeus*) avoiding males (Kunz et al., 2021; Scott et al., 2019, 2023), black howler monkeys (*Alouatta pigra*) show heightened vigilance (Treves et al., 2003), and Bornean orangutan mothers stay close to infants when males are present (Scott et al., 2023). In addition, living in large groups with low kinship coefficients, females from different maternal lineages often compete for breeding and rearing opportunities, so aggression from conspecifics also poses a threat to offspring (Clutton‐Brock & Huchard, 2013). Dominant females often display aggression toward the newborn of subordinate females (Lloyd & Rasa, 1989; Silk et al., 1981). Aggression and harassment directed at the offspring of rival females have significant fitness consequences for both mother and offspring (Stockley & Bro‐Jørgensen, 2011).

Maternal vigilance serves the function of monitoring and protecting offspring (Caro, 2005). In comparison to females without infants, females with infants have a greater need to monitor their surrounding environment as they must detect and avoid threats to themselves and their offspring (Treves et al., 2003). The optimal strategy for maternal monitoring behavior refers to the ability of mothers to make flexible adjustments based on the risks faced by their offspring (Lima & Bednekoff, 1999). Understanding the function of monitoring behavior usually comes from evaluating the circumstances in which it occurs (Gaynor & Cords, 2012). To test relevant hypotheses, it is necessary to correlate vigilance behavior with potential threats (Caro, 2005). However, little is known about how the effect of threats faced by infants modulates maternal visual monitoring and is influenced by the neighbors. Hence, we studied the visual monitoring behavior of females with infants in a free‐ranging group of Tibetan macaques (*M. thibetana*) in Huangshan, China.

Tibetan macaques represent an excellent model for studying the effect of social threat on visual monitoring. First, their habitat does not include large predators, and there are no records of individuals of this group being preyed on (Li, 1999). Thus, the vigilance of mothers was likely to be directed toward members within the group or offspring. Second, female macaques exhibit strong kin preferences concerning affiliation and tolerance (Berman et al., 2004) and relatives frequently provide support during conflicts and collectively monitor potential threats within the surrounding environment (Li et al., 2004; Tai et al., 2022), whereas such relationships between non‐kin individuals are rare. Thirdly, Tibetan macaques live in a multi‐male, multi‐female society characterized by strict linear hierarchies (Berman et al., 2004). Aggression directed by dominant individuals to subordinates is common, and subordinates are unlikely to win encounters with higher‐ranking competitors. Moreover, a previous study found that many of the infants were severely injured shortly before their deaths, indicating aggression by adults toward infants in Tibetan macaques (Berman et al., 2007). Therefore, females with infants need to monitor conspecifics.

To investigate the flexibility of maternal visual monitoring in response to infant risk perception, we proposed several predictions. First, the characteristics of the infant, such as age and sex, may also influence maternal visual monitoring. Accordingly, we predicted the maternal level of visual monitoring would decrease as the infant's age in months increased, and mothers with male infants would display higher levels of visual monitoring than mothers with female infants. Second, maternal characteristics may influence monitoring behavior. We predicted that the higher the maternal rank, the lower the level of visual monitoring, and the level of visual monitoring would be higher in the primiparous females than in the multiparous females. Additionally, we also predicted that maternal monitoring levels would increase as the distance between infants and mothers increased. Finally, social environment is also an important factor affecting maternal visual monitoring, neighbors with different characteristics may have different effects on the mother's visual monitoring. Thus, we predicted that the level of maternal visual monitoring would decrease as the number of kin increased, and increase as the number of non‐kin males/higher ranking non‐kin individuals increased.

## 2 MATERIALS AND METHODS

### 2.1 Study area and subjects

We studied a habituated group of Tibetan macaques (Group Yulinkeng 1 or YA1) inhabiting the “Valley of the Wild Monkeys” in Huangshan, China. More details about the study site can be found in Xia et al. (2012). Continuous research has been conducted on this group since 1986, during which characteristics and life history information of members within the group were recorded. The group consisted of 27 adult individuals during the study period, with 12 adult females raising infants (Table 1). The age range of Tibetan macaque infants is 0–12 months old (Li, 1999). However, during the study period, a female Tibetan macaque (YCH) continued to nurse and care for her infant (YXL) to 23 months old, until YCH gave birth to a new infant (YXS) in February 2023. Therefore, we also included YCH‐YXL as subjects in the study, despite YXL being over 12 months old (Table 1). Each member was individually recognized based on distinctive physical features such as scars, hair color patterns, and/or facial/body appearance (Li, 1999; Xia et al., 2012).

**TABLE 1:** Demographic structure of study animals in YA1 group during the observation.

Male			Female		
ID/Mother	Immigrate date	David's score	ID/Mother	Immigrate date	David's score
	Birth date			Birth date	
**Adults (12)**			**Adults (15)**		
YXK/YH	2013‐02‐14	178.56	YH/ 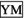	2003	78.20
TQS/TXH	2015‐05‐04	25.22	TH/ 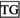	2003	−68.50
WM	2018‐11‐20 Immigrate	123.90	TXX/TH	2008‐03‐17	−96.19
TQ	2018‐11‐27 Immigrate	121.40	YCY/ 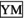	2009‐03‐12	17.47
NM	2021‐08‐08 Immigrate	78.60	THY/TR	2009‐04‐08	−106.33
YL	2021‐08‐24 Immigrate	203.11	TXH/TH	2009–2010	−23.51
DB	2021–09 Immigrate	−25.28	YXX/YH	2010‐05‐08	101.97
QT	2021‐09‐05 Immigrate	−69.64	THX/TR	2012‐04‐19	−155.40
BHZ	2021‐10‐15 Immigrate	2.37	YCL/ 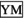	2012‐09‐15	−27.17
DZ	2021‐11‐03 Immigrate	−24.20	TQL/TXX	2013‐03‐25	−80.09
WS	2022‐01‐04 Immigrate	−20.14	YXY/YH	2015‐04‐19	17.60
LB	2021‐11‐22 Immigrate	−22.16	YCH	2015‐10 Immigrate	−13.32
			TQY/TXX	2016‐03‐04	−116.30
			TFH/THY	2016‐03‐27	−80.28
			TQG/TXH	2017‐06‐18	−40.01
**Inf (7)**			**Inf (7)**		
TDQ/TQL	2022‐02‐20		YXL/YCH	2021‐03‐30	
TXL/TH	2022‐03‐03		TFR/THY	2022‐02‐27	
YXH/YH	2022‐03‐20		TQR/TXX	2022‐04‐25	
TFA/THX	2022‐04‐11		YXR/YCY	2022‐04‐30	
TDD/TQY	2022‐04‐11		YQK/YXX	2022‐05‐30	
YQF/YXY	2022‐04‐25		YXW/YCL	2022‐5	
YXS/YCH	2023‐02‐11		TQK/TXH	2022‐06‐25	

*Note*: Individuals in the box (e.g., YM) mean the individuals died before the study was conducted. “Immigrate” means the individual was an immigrant. TH's infant TXL during the study period was picked up and raised by TQL shortly after TXL's birth (see: Wu et al., 2023), so we did not include TH as a mother.

### 2.2 Data collection

Behavioral observations were conducted between August 2022 and February 2023. During the study period, we used a DV camera (FDR‐APX55; Sony Corporation, Tokyo, Japan) to record all behavioral data of subjects from 8:00 to 17:00. We employed a 10‐min focal animal sampling method and continuous recording to observe the 12 females with infants (Altmann, 1974). The observation order of each focal individual was determined randomly. If the focal subject could not be found, we selected the next subject on the list and returned to the previous female when it reappeared (Xia et al., 2012). Each individual was observed only once per day. During the study, we conducted 45 days of effective observation on 12 subjects, resulting in a total of 192 focal samples collected over a total duration of 35.54 h. The average observation time per individual was 164.04 min (SD: 2.99 min).

Ad libitum sampling was performed to record data on agonistic (aggressive/submissive) interactions between adults. Aggressive interactions were scored when one individual threatened, chased, slapped, grabbed, or bit another individual (Berman et al., 2004). Submissive interactions were defined as behaviors in which individuals showed fearful reactions, such as avoiding and fleeing (Berman et al., 2004). When subjects were involved in conflicts, we promptly recorded the identities of the aggressor and victim. David's score (DS) was calculated to determine the dominant rank among individuals (more details see: Gammell et al., 2003). DS reflects the individual's dominant rank, with higher scores indicating higher rank (Table 1).

**FIGURE 1:**
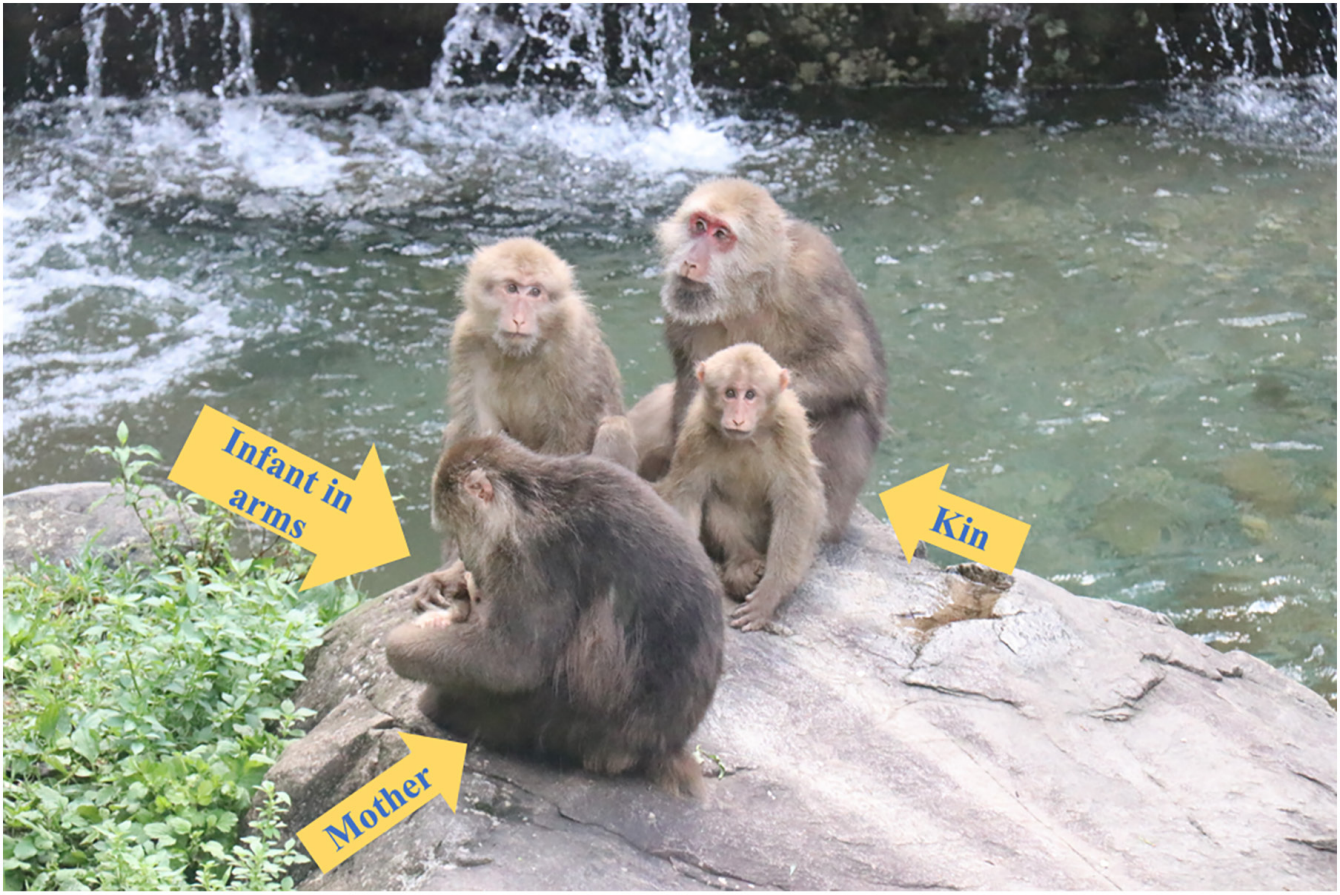
Visual monitoring of Tibetan macaque.

Visual monitoring (Figure 1) was defined as any scanning directed beyond the arm's reach of the focal animal (Allan & Hill, 2018; Treves, 2000). First, to collect data on maternal visual monitoring, we ensured that the faces or eyes of the subjects were visible during observation. When it was impossible to directly observe the focal subject's eyes or face, we assessed the maternal visual monitoring based on the movements of her head or neck (Kutsukake, 2006). Second, based on the studies of others (Iki & Kutsukake, 2021; McDougall & Ruckstuhl, 2018), we define the beginning of visual monitoring as when the individual looks up and toward the outside world and the end as when the individual looks down and toward the arm. Third, although visual monitoring may also serve other functions such as searching for food or planning movement routes, it is focused on monitoring a specific individual in terms of external vigilance (Treves et al., 2001), and it suggests that visual monitoring also serves as a means for risk detection (Gaynor & Cords, 2012). Finally, due to challenges in identifying specific targets for monitoring under field conditions, we did not attempt to differentiate between monitoring the surrounding environment and monitoring other group members. Events of visual monitoring were recorded for all focal individuals who met these above criteria.

During the observation period, we recorded the distance between the mother and infant, as well as the identity of neighbors within 5 m of the adults. We quantified the social environment of focal subjects based on mother–infant distance and neighbor identities. Spatial relationships are particularly sensitive to proximal threats and often reflect maternal protectiveness (Fairbanks, 1996). Therefore, we used the distance between a mother and her infants as an index of the amount of protection or attention given by a mother to her dependent offspring (Cameron et al., 2003). We categorized the distance between a mother and her infant into three categories: within reach (0–1 m), nearby (1–5 m), and beyond nearby (>5 m). Meanwhile, we used matrilineality information from 27 adults, as well as known maternal kinship relations (mother/offspring, maternal siblings, and grandmother/grandchild). Adults belonging to these matrilineal kinship categories were considered kin, while others were considered non‐kin. And “non‐kin male” refers to males lacking kinship relations, “higher‐ranking non‐kin” refers to higher‐ranking individuals lacking kinship relations.

The focal session was composed of different social environments. When there was a change in the distance between mother and infant or a change in the composition/number of neighbors, we considered it as a new social environment. Therefore, the composition/number of neighbors and the mother–infant distance did not change within a single social environment (Iki & Kutsukake, 2021). We recorded the total duration of time spent in a specific social environment.

In our study, we used the proportion of time spent on visual monitoring and the frequency of visual monitoring as indicators of maternal visual monitoring level. The proportion of time was the total duration of time spent in a specific social environment phase. The frequency was the number of visual monitoring bouts in a specific social environment phase. These two indicators are often used to study the vigilance of animals (the proportion of time spent vigilant: Kutsukake, 2006; Busia et al., 2019; Iki & Kutsukake, 2021; the frequency of vigilance: Teichroeb & Sicotte, 2012; Iki & Kutsukake, 2021; for a review, see Allan & Hill, 2018).

### 2.3 Data analysis

We ranked the aggression–submission bouts of adults to determine their rank order of dominance. The number of wins and losses in conflicts was put in a paired interaction matrix, and the rank order was determined by minimizing the number of wins below the diagonal. Based on the index of aggression‐submission behavior and the win–lose ratio. We calculated the dominance hierarchy based on aggressive–submissive behavior and the weights of wins and losses between individuals, by using an aggressive–submissive matrix to compute DS (Gammell et al., 2003). During the observation period of this study, a total of 761 pairs of aggressive‐submissive behaviors were recorded for all individuals in the Tibetan macaque population.

To examine the influence of maternal characteristics, infant characteristics, and social environment on the proportion of time spent on maternal visual monitoring. We fitted a generalized linear mixed model (GLMM) with a Gamma error structure and a log link function using the glmer function in the lme4 package in R version 4.2.3 (R Core Team, 2023). For the analysis of visual monitoring frequency, we applied a GLMM with a Poisson error structure and log link function. The offset term of all models was the log‐transformed value of the total duration of time spent in a specific social environment phase.

First, we included maternal parity and maternal rank as fixed effects in the model to examine the effect of maternal characteristics on maternal visual monitoring levels. Second, to assess the influence of infant characteristics on maternal visual monitoring levels, we added infant age in months and infant sex as fixed effects in the model. Furthermore, to investigate the effect of the social environment on maternal visual monitoring levels, we included the number of kin, the number of non‐kin males, and the number of higher‐ranking non‐kin individuals in the neighborhood as fixed effects in the model. Additionally, to address issues of pseudoreplication with focal sampling data, we included the phases and identities of individuals as random effects in the model. Finally, we assessed the degree of correlation between the independent variables by conducting a variance inflation factor (VIF) test on the model, with a threshold set at 5.

We used an information criterion‐based multimodel inference method to account for the uncertainty in model selection (Burnham & Anderson, 2002). First, we employed the dredge function in the MuMln package to calculate and compare Akaike information criterion (AICc) scores for all possible combinations of fixed effects in all models. Although the model with the lowest AICc score was considered the best model that provides a satisfactory explanation of the variation in the data, models with a ΔAIC < 2 are considered to have a similar level of statistical support as the best model (Burnham & Anderson, 2002). Subsequently, we computed weights (*wi*) for each ΔAIC<2 models. If the weight of the best model is <0.9, indicating uncertainty in model selection, we employed the model. avg function in the MuMln package to conditionally average all models with ΔAIC < 2. This approach allows us to thoroughly consider the effect sizes of predictors in the models. (Brand & Marchant, 2018; Burnham & Anderson, 2002). All data analyses were performed using R version 4.2.3 (R Core Team, 2023).

### 2.4 Ethics statement

The study was completely observational and did not affect the welfare of the Tibetan macaques. This study was conducted with the Huangshan Monkey Management Center and the Huangshan Garden Forest Bureau. It complies with the regulations of the Chinese Wildlife Conservation Association regarding the ethical treatment of research subjects, and under the law of the People's Republic of China on the protection of wildlife.

## 3 RESULTS

### 3.1 The proportion of time spent on maternal visual monitoring

**FIGURE 2:**
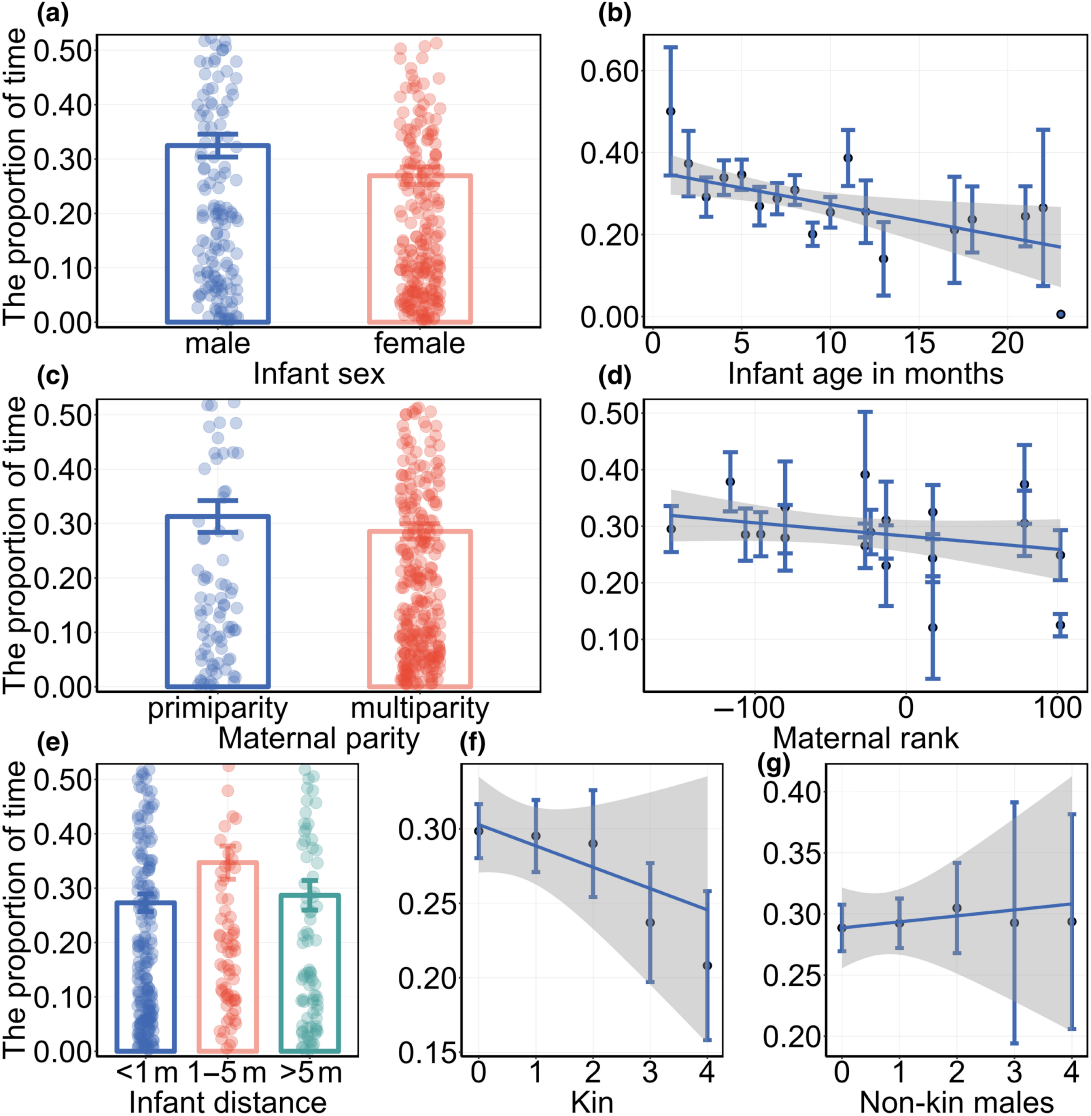
The proportion of time spent on maternal visual monitoring according to the fixed effects (a) infant sex, (b) infant age in months, (c) maternal parity, (d) maternal rank based on Davids's score, (e) the distance between mother and their infant, (f) the number of nearby kin, (g) the number of nearby non‐kin males. In (a, c, e) the points are the jitter of the sample, the error bars represent the standard deviation (SE). In (b, d, f, g) the black dots represent the mean values, the error bars represent the standard deviation (SE), the shaded areas represent the 95% confidence intervals.

For the proportion of time spent on maternal visual monitoring multi‐model analysis, we weighted the average of all models with ΔAIC < 2 based on their weights (Table 2), and the results (Table 3) showed that the characteristics of the infant influenced the proportion of time spent on maternal visual monitoring. Females with male infants spent a greater proportion of time than mothers with female infants (*β* ± SE = 0.152 ± 0.095, Figure 2a), and the proportion of time decreased as infants aged (*β* ± SE = −0.031 ± 0.013, Figure 2b). Maternal characteristics influenced the proportion of time spent on maternal visual monitoring. Multiparous females spent less time monitoring than primiparous females (*β* ± SE = −0.173 ± 0.112, Figure 2c), females with higher dominant rank spent less time on visual monitoring than lower ranked females (*β* ± SE = −0.001 ± 0.001, Figure 2d).

**TABLE 2:** The proportion of time models with ΔAIC < 2 were selected for model selection.

Model	df	logLik	AICc	ΔAIC	*w*_*i*_
INA + INS	6	−2053.748	4119.694	0	0.078
MID + INA + INS + KIN	9	−2050.750	4119.926	0.232	0.070
INA + INS + KIN	7	−2052.858	4119.979	0.285	0.068
INA + MOP	6	−2053.953	4120.102	0.408	0.064
INA + KIN+MOP	7	−2052.966	4120.195	0.501	0.061
MID + INA + INS	8	−2051.988	4120.316	0.622	0.057
INA	5	−2055.140	4120.421	0.727	0.054
MID + INA + KIN	8	−2052.080	4120.499	0.805	0.052
MID + INA	7	−2053.255	4120.774	1.080	0.046
INA + KIN	6	−2054.294	4120.785	1.091	0.045
INA + INS + MOP	7	−2053.343	4120.950	1.256	0.042
MID + INA + KIN+MOP	9	−2051.281	4120.988	1.294	0.041
INA + INS + KIN+MOP	8	−2052.365	4121.070	1.375	0.039
INA + INS + MOR	7	−2053.504	4121.272	1.578	0.036
INS	5	−2055.605	4121.351	1.657	0.034
MID + INA + MOP	8	−2052.550	4121.439	1.745	0.033
INA + MOR	6	−2054.651	4121.500	1.806	0.032
INA + KIN+MOP+NKM	8	−2052.626	4121.592	1.898	0.030
INA + INS + NKM	7	−2053.675	4121.614	1.920	0.030
MID + INA + INS + KIN+MOP	10	−2050.553	4121.627	1.933	0.030
MID + INA + INS + KIN+NKM	10	−2050.556	4121.633	1.939	0.030
MID + INA + INS + KIN+MOR	10	−2050.566	4121.654	1.960	0.029

*Note*: Models with ΔAIC > 2 are not presented.

Abbreviations: AICc, Akaike information criterion corrected value; INA, infant age in month; INS, infant sex; KIN, the number of kin; logLik, log‐likelihood value; MID, mother–infant distance; MOP, maternal parity; MOR, maternal rank; NKH, the number of higher‐rank non‐kin individuals; NKM, the number of non‐kin males; *w*_*i*_, Akaike weight; ΔAIC, difference between the AICc value of the model and the optimal model AICc value.

**TABLE 3:** Details of the model averaging for the proportion of visual monitoring time.

Factors	Estimate (*β*)	Adjusted SE	VIF
Intercept	−0.978	0.230	
INA	−0.031	0.013	1.374
INS: male	0.152	0.095	1.851
KIN	−0.080	0.050	1.171
MID: within reach	−0.231	0.117	2.171
MID: beyond nearby	−0.175	0.141	2.171
MOP: multi	−0.173	0.112	1.130
MOR	−0.001	0.001	1.048
NKM	0.036	0.037	1.109

*Note*: The estimated values indicated the magnitude and direction of the impact of each condition on the dependent variable (the proportion of time spent on maternal visual monitoring), with participant identity included as a random factor.

Abbreviations: INA: infant age in month; INS: infant sex; KIN: the number of kin; MID: mother–Infant distance; MOP: maternal parity; MOR: maternal rank; NKM: the number of non‐kin male; VIF: variance inflation factor.

The distance between the mother and infant was also an important factor that affected the proportion of time spent on maternal visual monitoring. When the infant was nearby, the mother spent a higher proportion of time compared to when the mother–infant distance was within reach (*β* ± SE = −0.231 ± 0.117, Figure 2e) and beyond nearby (*β* ± SE = −0.175 ± 0.141, Figure 2e). Finally, we found that the number of neighbors with different characteristics affected the proportion of time spent on maternal visual monitoring. The proportion of time decreased with an increase in the number of kin among neighbors (*β* ± SE = −0.080 ± 0.050, Figure 2f). Conversely, it increased with an increase in the number of non‐kin male individuals among neighbors (*β* ± SE = 0.036 ± 0.037, Figure 2g).

### 3.2 The frequency of maternal visual monitoring

**FIGURE 3:**
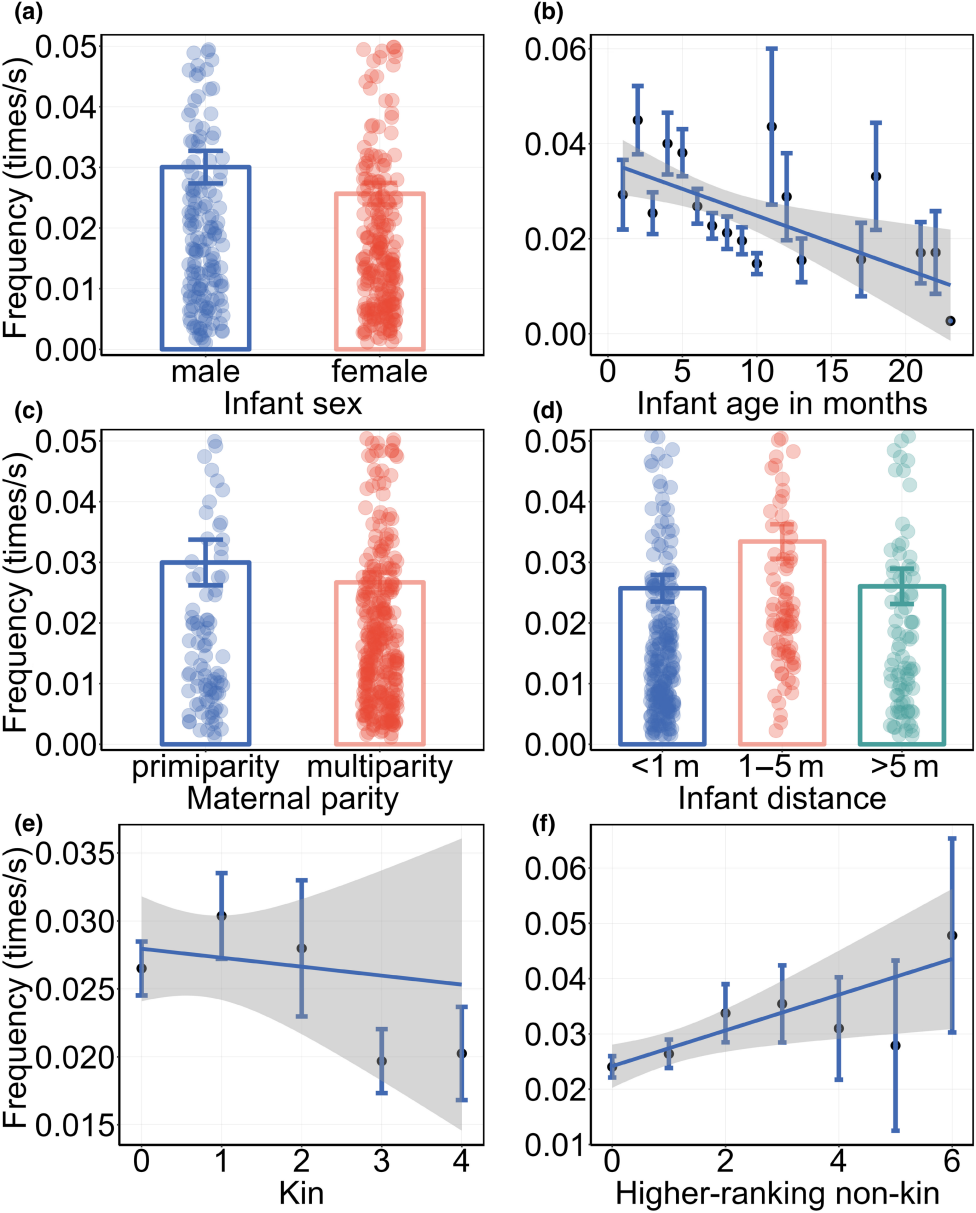
The frequency of maternal visual monitoring according to the fixed effects (a) infant sex, (b) infant age in months, (c) maternal parity, (d) the distance between mother and their infant, (e) the number of nearby kin, (f) the number of higher‐ranking non‐kin neighbors. In (a, c, d) the points are the jitter of the sample, the error bars represent the standard deviation (SE). In (b, e, f) the black dots represent the mean values, the error bars represent the standard deviation (SE), the shaded areas represent the 95% confidence intervals.

For the frequency of maternal visual monitoring multi‐model analysis, we also weighted the average of all models with ΔAIC < 2 based on their weights (Table 4), and the results (Table 5) showed that mothers with male infants had a higher monitoring frequency than mothers with female infants (*β* ± SE = 0.053 ± 0.057, Figure 3a), as the infant age in months increased, the maternal monitoring frequency decreased (*β* ± SE = −0.046 ± 0.009, Figure 3b). And maternal parity influenced her monitoring frequency. Compared to primiparous females, multiparous females had a higher monitoring frequency (*β* ± SE = 0.030 ± 0.087, Figure 3c). The characteristics of the infant impacted the maternal monitoring frequency.

**TABLE 4:** The frequency models with ΔAIC < 2 were selected for model selection.

Model	df	logLik	AICc	ΔAIC	*w*_*i*_
MID + INA	6	−999.158	2010.513	0	0.354
MID + INA + INS	7	−998.735	2011.734	1.221	0.192
MID + INA + NKH	7	−998.899	2012.061	1.548	0.163
MID + INA + KIN	7	−998.939	2012.142	1.629	0.157
MID + INA + MOP	7	−999.101	2012.466	1.953	0.133

*Note*: Models with ΔAIC > 2 are not presented.

Abbreviations: AICc, Akaike information criterion corrected value; INA, infant age in months; INS, infant sex; KIN, the number of kin; logLik, log‐likelihood value; MID, mother–infant distance; MOP, maternal parity; MOR, maternal rank; NKH, the number of higher‐ranking non‐kin individuals; *w*_*i*_, Akaike weight; ΔAIC, difference between the AICc value of the model and the optimal model AICc value.

**TABLE 5:** Details of the model averaging for the frequency of visual monitoring.

Factors	Estimate(*β*)	Adjusted SE	VIF
(Intercept)	−3.709	0.123	
INA	−0.046	0.009	1.564
MID: within reach	−0.337	0.079	1.060
MID: beyond nearby	−0.301	0.092	1.060
INS: male	0.053	0.057	1.241
NKH	0.019	0.026	1.040
KIN	−0.021	0.032	1.085
MOP: multi	0.030	0.087	1.692

*Note*: The estimated values indicated the magnitude and direction of the impact of each condition on the dependent variable (the frequency of maternal visual monitoring), with participant identity included as a random factor.

Abbreviations: VIF: variance inflation factor. MID: mother–infant distance; INA: infant age in months; INS: infant sex; MOP: maternal parity; MOR: maternal rank; KIN: the number of kin, NKH: the number of higher‐ranking non‐kin individuals.

The maternal monitoring frequency was influenced by the mother–infant distance, the mother showed a higher frequency when the infant was nearby compared to when the mother‐infant distance was within reach (*β* ± SE = −0.337 ± 0.079, Figure 3d) and beyond nearby (*β* ± SE = ‐0.301 ± 0.092, Figure 3d). Finally, the number of neighbors with different characteristics affected the maternal visual monitoring frequency. As the number of kin increased, the maternal monitoring frequency decreased (*β* ± SE = −0.021 ± 0.032, Figure 3e). Conversely, with an increase in the number of higher‐ranking non‐kin individuals, the maternal monitoring frequency increased (*β* ± SE = 0.019 ± 0.026, Figure 3f).

## 4 DISCUSSION

In this study, we investigated the adaptive monitoring strategies of a group of free‐ranging female Tibetan macaques with infants in response to perceived threats within their social environment. We employed a GLMM to examine the influence of maternal characteristics, infant characteristics, and mother–infant distance. Furthermore, we explored how risks posed by group members toward a mother and her infant could serve as a strong predictor for the level of visual monitoring exhibited by the mother.

Our results support our prediction that infant characteristics would influence mothers' level of visual monitoring. Mothers reduce their investment in offspring as they become more independent with age (Soben et al., 2023). This pattern is also observed in Tibetan macaques, where the proportion of time and frequency of maternal visual monitoring decreases as infant age increases, consistent with previous studies on mantled howler monkeys showing a decrease in maternal vigilance as offspring grow older (Dias et al., 2018). In addition, our results found that mothers with male infants had higher levels of visual monitoring than mothers with female infants. For macaques, sex‐specific maternal care may be attributed to the fact that males emigrate to join new troops during puberty and must be physically healthy enough to be accepted (Dettmer et al., 2016). In rhesus macaques (*Macaca mulatta*), mothers spend more time caring for male infants, which improves their chances of survival which may help the males immigrate a new group as adults (Hinde, 2007; Hinde et al., 2015).

We observed that the distance between the mother and infant influenced the level of maternal visual monitoring. When the infants began to actively move away from their mothers and explore the surroundings, this period was characterized by heightened vigilance on the side of mothers (Simpson, 1988), as it represented an increased potential risk for the infants while separating from their mothers (Maestripieri, 1993; Onishi & Nakamichi, 2011; Otali & Gilchrist, 2006). Furthermore, staying close between mother and infant is generally considered a protective strategy against infanticide for mothers (Palombit, 2012). Our findings partially supported the prediction of mother–infant distance, since maternal monitoring levels were higher when infants were 1–5 m compared to when they were < 1 m of mothers. In chimpanzees and Japanese macaques increased maternal vigilance time or monitoring frequency during periods of separation between infants and mothers (Kutsukake, 2007; Onishi & Nakamichi, 2011). In our study, Tibetan macaque infants were separated but still near their mothers, they may have been closer to neighboring individuals than their mothers. Consequently, this could have led to heightened vigilance of mothers. Our finding was supported by another research among chimpanzees, when there were neighbors and some threats from conspecific, infant separation increased maternal vigilance, but when there were no neighbors and a high predation risk, infant separation did not increase vigilance (Kutsukake, 2006).

However, when the infant was too far from its mother, >5 m, the mother reduced her visual monitoring time and frequency, not supporting our prediction. There may be two reasons for the result. Mothers trade off vigilance with other kinds of behaviors like grooming (Blanchard et al., 2017; Onishi & Nakamichi, 2011). For example, rhesus macaque mothers spend more time grooming when infants move away, and we may be observing a similar pattern in Tibetan macaques (Maestripieri, 1993). Alternatively, mothers may reduce monitoring when the risk to infants is low, regardless of the distance that infants travel from the mother.

We predicted that primiparous females would exhibit higher visual monitoring levels than multiparous females in Tibetan macaques. Our research supported that, in contrast to multiparous females, primiparous females spent more time monitoring their surroundings and had lower monitoring frequencies. These results suggested that a mother's reproductive experience influences her visual monitoring strategy, with primiparous mothers engaged in long‐time and low‐frequency monitoring while multiparous mothers adopted short‐time and high‐frequency vigilance strategies. Similarly, in chimpanzees (*Pan troglodytes*), primiparous mothers invest in care behaviors differently than more experienced mothers (Stanton et al., 2014). In humans (*Homo sapiens*) primiparous mothers engage in more social and caretaking behavior with their first child than with their second child (Jacobs & Moss, 1976).

Our results supported the prediction, the maternal visual monitoring of Tibetan macaques living in a despotic society would be influenced by dominance rank since mothers with lower rank had higher monitoring levels, consistent with other reports that low‐ranked individuals are more vigilant than higher‐ranking individuals (Blumstein et al., 2001; Pannozzo et al., 2007; Shepherd et al., 2006). This finding may be explained by lower‐ranking individuals are likely to face social threats more frequently than higher‐ranking individuals and they suffer from greater social risk. Hence, lower‐ranking mothers may respond to numerous threats by engaging in higher monitoring levels (Iki & Kutsukake, 2021). Similarly, the follow‐up analyses also suggested mothers increased attention to higher‐ranking individuals. We found that as the number of higher‐ranking non‐kin individuals nearby increased, so did the frequency of maternal visual monitoring. Higher‐ranking non‐relatives not only pose a threat to the mother but also to infants. The aggressive behavior of dominant individuals usually reduces direct benefits (e.g., space or resources) of breeding females and their offspring (Rödel et al., 2008; Tuomi et al., 1997), it may generate strategic benefits by limiting future resource competition or contributing to the maintenance of dominant mothers' social status or territory (Digby, 2000). However, the exact reason for the increase in maternal visual monitoring levels is unclear, whether it is due to mothers protecting themselves or their offspring, or both. Therefore, future research needs to be more specific.

Maternal vigilance is also associated with the risks posed by non‐kin males within the group. Therefore, we predicted that maternal monitoring levels would increase in the presence of non‐kin males. Our findings supported this prediction, as maternal visual monitoring increased, the number of non‐kin males among neighbors increased. Previous studies have also reported mothers maintain closer proximity to their offspring and exhibit increased vigilance levels when the presence of males (Otali & Gilchrist, 2006; Treves et al., 2003). Previous research by Berman et al. (2007) suggested that adults in Tibetan macaques would attack infants, but there was a lack of evidence as to which group members were responsible for such attacks. Our study provides indirect evidence that female macaques employ strategies consistent with avoiding infanticide, by increasing their vigilance levels to monitor nearby non‐kin males and high‐ranking non‐kin individuals.

Additionally, our findings indicate a negative correlation between the mother's level of monitoring and the number of kin, supporting our prediction. Tibetan macaques exhibit strong preferences for affiliation and tolerance toward their kin (Berman et al., 2004). Kin selection theory (Hamilton, 1964) suggests that competition between relatives should be less intense than between unrelated females, female kin commonly associate with and support each other (Clutton‐Brock & Huchard, 2013). It means that kin may serve as potential allies, allowing individuals to rely on the vigilance of kin to reduce their vigilance (Iki & Kutsukake, 2021). Our study provides further support for this argument and was consistent with previous research demonstrating decreased vigilance when in the presence of kin or constitute the majority nearby (Busia et al., 2019; Gaynor & Cords, 2012; Iki & Kutsukake, 2021).

Strategies adopted by females in response to social risks from males can generally be categorized into three types (Palombit, 2015): (1) sexual behavior and reproduction, for example, promiscuity/multimale mating (Bertram, 1975; Dugdale et al., 2011). (2) Individual counterstrategies, for example, maternal aggression (Oleinchenko, 2012), and visual monitoring (Cameron et al., 2003). (3) Social counterstrategies, for example, association (Opie et al., 2013; Rémy et al., 2013) and coalitionary defense (Cords & Fuller, 2010). The above results indicate that in Tibetan macaques, females with infants can employ individual/social counterstrategies to protect their infants, such as monitoring potentially risky individuals or forming alliances with kin in neighbors.

In conclusion, our study highlights the utilization of counterstrategies by female Tibetan macaques to mitigate the risks to infants and enhance protection for their infants. This study contributes to our understanding of how social factors influence the level of visual monitoring performed by Tibetan macaque mothers, reflecting their ability to flexibly adjust monitoring strategies in response to social risks. However, maternal protective strategies are not limited to a single approach but encompass a series of strategies (Palombit, 2015). Therefore, it is necessary to attribute maternal protective strategies to multiple factors, including quantifying the specific social risks faced by individuals and evaluating the trade‐offs that mothers engage in various adaptive behaviors.

## AUTHOR CONTRIBUTIONS

**Shen‐Qi Liu:** Conceptualization (equal); data curation (lead); formal analysis (lead); investigation (lead); methodology (equal); visualization (lead); writing – original draft (lead); writing – review and editing (lead). **Hong‐Wei Tian:** Conceptualization (supporting); investigation (equal); writing – review and editing (supporting). **Sen Ren:** Investigation (supporting); writing – review and editing (supporting). **Wen‐Kai Sun:** Investigation (supporting); writing – review and editing (supporting). **Peng‐Lai Fan:** Conceptualization (supporting); investigation (supporting); writing – review and editing (supporting). **Dong‐Po Xia:** Conceptualization (supporting); investigation (supporting); writing – review and editing (supporting). **Bing‐Hua Sun:** Conceptualization (supporting); investigation (supporting); writing – review and editing (supporting). **Jin‐Hua Li:** Conceptualization (supporting); formal analysis (equal); writing – review and editing (equal). **Xi Wang:** Conceptualization (lead); funding acquisition (lead); resources (lead); supervision (equal); visualization (equal); writing – original draft (equal); writing – review and editing (equal).

## FUNDING INFORMATION

National Natural Science Foundation of China, Grant/Award Number: 31801983, 31971404, 32370535; Key Project for Natural Science Research in Universities of Anhui Provincial Department of Education, Grant/Award Number: 2022AH050114.

## CONFLICT OF INTEREST STATEMENT

None declared.

## Data Availability

The dataset generated and analyzed during the current study is available in the open figshare repository, https://doi.org/10.6084/m9.figshare.25124042.
